# Oral health-related quality of life in adult inpatients with psychiatric and/or substance use disorders: a Norwegian cross-sectional study

**DOI:** 10.3389/froh.2026.1805653

**Published:** 2026-05-04

**Authors:** Kristina G. Kantola, Rolf Wynn, Hege Nermo, Jan-Are Kolset Johnsen, Elin Hadler-Olsen

**Affiliations:** 1 The Public Dental Health Service Competence Center of Northern Norway, Tromsø, Norway; 2 Department of Clinical Medicine, Faculty of Health Sciences, UiT The Arctic University of Norway, Tromsø, Norway; 3 Department of Clinical Dentistry, Faculty of Health Sciences, UiT The Arctic University of Norway, Tromsø, Norway; 4 Department of Medical Biology, Faculty of Health Sciences, UiT The Arctic University of Norway, Tromsø, Norway

**Keywords:** general health, mental disorders, oral health, oral health related quality of life, orofacial pain, psychiatry, psychosis, substance use

## Abstract

**Introduction:**

This study aimed to assess the prevalence and nature of oral health impacts on daily life and examine the relationship between psychiatric symptoms, oral health problems, and oral health related quality of life (OHRQoL) in individuals with severe psychiatric and/or substance use disorders.

**Methods:**

Data were collected from a cross-sectional study of 138 adult inpatients (median age 35.5 years, range 19–70, 60% men, 40% women) at the Division of Mental Health and Substance Use, University Hospital of Northern Norway. Participants underwent clinical oral examinations including radiographs. A structured questionnaire covered various health domains, including oral health. The primary outcome was OHRQoL, assessed by the Oral Impact on Daily Performance (OIDP) questionnaire. Data were analyzed with descriptive statistics, cross-tabulations, regression analysis, and Hayes' PROCESS Macro v4.2 (Model 4) for simple mediation analyses.

**Results:**

Problems with at least one of the activities assessed with the OIDP questionnaire were reported by 72.3% of the participants with more than 60% having issues at least weekly. Most common were problems with eating (54.4%), cleaning- or showing teeth (40.0%), and sleeping (35.8%). Mediation analyses showed that general health, psychological distress, and (prodromal or current) psychosis symptoms were directly linked to OIDP. Tooth decay and missing teeth were independently associated with OIDP, while subjective symptoms including orofacial pain and xerostomia showed patterns consistent with indirect associations.

**Discussion:**

This study found a strong association between oral problems and quality of life among adults facing severe psychiatric and/or substance use disorders. The activities most often affected by oral problems were eating, showing teeth, tooth cleaning and sleeping, which are important for both physical and mental health. These findings underscore the importance of integrated, interdisciplinary oral health care in psychiatric- and substance use services to address pain and tooth loss to improve quality of life.

## Introduction

Severe substance use disorders or severe psychiatric illnesses, that often require specialized or inpatient care, increase the risk of oral health problems (1, 2). Affected individuals frequently experience poor general health and are disproportionately affected by socioeconomic challenges (3, 4), including limited educational attainment (5–7), economic hardship (4), unemployment (8) social exclusion (9–11) and a substantially reduced life expectancy (12–14). Severe mental illness and substance use are commonly associated with behavioral risk factors such as poor oral hygiene, frequent consumption of sugary substances, smoking, and bruxism (2). These behaviors, combined with limited access to preventive care, increase the likelihood of developing caries, tooth loss, and orofacial pain (2).

Polypharmacy is common in both psychiatric and addiction treatment. Many prescribed medications cause xerostomia (dry mouth). Xerostomia may result from the medications' antimuscarinic effects and their tendency to shift the autonomic balance toward sympathetic predominance, thereby reducing salivary secretion (15). This may complicate eating, impair self-cleaning of the mouth, and further increase caries risk (16, 17).

Despite well-documented oral health disparities in individuals with substance use disorders and psychiatric illness, most research has focused on clinical indicators such as decayed, missing, and filled teeth (DMFT). While some studies have addressed oral health–related quality of life, relatively few have examined how oral conditions affect daily functioning, social interactions, or psychological well-being from the individual's perspective among individuals receiving inpatient treatment for severe psychiatric and/or substance use disorders.

A classical disability framework has been articulated by WHO (ICIDH/ICF) (18, 19), Fisher-Owens (20), and Locker (21), which propose a hierarchical sequence linking health conditions to their social and functional consequences. These models conceptualize health outcomes as progressing from disease to impairment, which subsequently may lead to functional limitation, disability and reduced quality of life. Oral Health-Related Quality of Life (OHRQoL) is a concept that captures the subjective burden of oral conditions on individuals' daily lives. It includes psychological, physical, and social well-being, and within this disability framework, OHRQoL is understood as the endpoint of a causal chain in which biological, psychological, and behavioral factors interact.

The role of psychiatric symptoms, such as anxiety and depression, in shaping oral health experiences is not well understood. Within the disability model, psychiatric disorders, substance use disorders and general health problems, can be viewed as the disease-level factors, representing underlying conditions that can influence oral health both directly and indirectly through behavioral pathways. While psychiatric symptom scales (HSCL-10, PQ-16) capture specific dimensions of mental health, self-reported general health reflects a broader perception of overall health status and underlying disease burden. Clinical oral measures such as decayed teeth (DT), missing teeth (MT), orofacial pain, and xerostomia align with the impairment level, capturing the functional and structural disruptions in oral health that may arise from disease. These impairments reflect the tangible oral consequences experienced by individuals with psychiatric disorders and/or substance use disorders. OHRQoL measured through instruments such as the Oral Impacts on Daily Performance (OIDP) (22) corresponds to the functional limitation/disability level, as it captures how oral impairments affect essential daily activities. Positioning the study variables within this established theoretical framework provides a coherent rationale for examining how disease-related factors may be associated with oral impairments, and further how these impairments translate into reduced oral health–related quality of life.

Oral health-related quality of life (OHRQoL) is particularly relevant in psychiatric inpatient populations, where individuals often experience a high burden of psychiatric symptoms, functional impairment, and social vulnerability. In this context, oral conditions may have a disproportionate impact on essential daily activities such as eating, sleeping, communication, and social interaction. Clinical indicators alone may therefore fail to capture the full extent of oral health burden, highlighting the importance of incorporating patient-reported outcomes such as OHRQoL.

Although oral health disparities in individuals with psychiatric and/or substance use disorders are well documented, studies examining OHRQoL in inpatient populations remain limited. In particular, few studies have explored how clinical oral conditions and psychiatric symptoms jointly relate to OHRQoL within a coherent theoretical framework.

This study aimed to assess the prevalence and nature of oral health impacts on daily life among individuals with severe psychiatric and/or substance use disorders and further seek to identify how different oral health problems and psychiatric symptoms relate to OHRQoL. By integrating clinical oral measures, psychological factors, and a disability-based conceptual model, the present study provides a more comprehensive understanding of how oral health problems translate into functional limitations and reduced quality of life in a vulnerable and understudied population.

## Material and methods

### Study design and recruitment

Data for this cross-sectional study were collected among inpatients in the Division of Mental Health and Substance Use at the University Hospital of North Norway between October 2021 and July 2023, as previously described (23). Participants completed a structured questionnaire and underwent clinical and radiological oral examinations.

Recruitment targeted adults aged 18 or older from specific units, including the emergency psychiatric wards, security wards, the ward for co-occurring substance use and severe mental disorders, and the substance use treatment wards. Individuals under 18, those admitted to gerontopsychiatric ward, or those unable to provide informed consent were not included in the study.

Information about the study was shared by ward staff, posters in common areas, and flyers placed on tables. Healthcare workers invited eligible patients to join the study. Patients who were interested received clear written and verbal information from dental assistants and signed a consent form before participating. They were also offered free dental cleaning and a gift card valued at 200 NOK (€17) as an incentive. All participants were admitted to the hospital due to severe substance use disorders and/or severe psychiatric illnesses, representing a clinically complex and severely affected inpatient population. Admission to inpatient care generally reflects severe clinical impairment, often involving an inability to care for oneself and/or risk of harm to oneself or others. In contrast, individuals receiving outpatient care typically present with less severe and more stable conditions. At the time of inclusion, patients were considered clinically stable and able to participate.

### Procedures

One trained dentist conducted all clinical oral examinations, which were supplemented with clinical photographs (Camera: Nikon D7000, Lense: Nikon SWM VR ED IF Micro 1:1 d62 Nano Crystal Coat/AF-S Micro Nikkor 105 mm 1:2.8G ED), as well as four bitewing. Two trained assistants conducted structured interviews using standardized questions, ensuring neutrality and providing clarification to avoid misinterpretation.

The clinical oral examiner knew that the participants were inpatients receiving treatment for substance use and/or psychiatric disorders but was effectively blinded to their specific diagnoses. In Norway, dental and medical records are maintained in separate systems, and no information regarding psychiatric diagnoses was available to the examiner. In addition, questionnaire data were collected and recorded by research personnel and were not accessible to the examiner at the time of the clinical assessment. These procedures minimized the risk of observational bias.

### Quality controls

Intra rater reliability for caries detection was good, with ICC values of 0.875 (95% CI 0.862–0.888) for primary caries and 0.783 (95% CI 0.759–0.805) for secondary caries. Quality control procedures including radiographic standardization, examiner calibration, and intraexaminer reliability assessment have been described in detail previously (23).

### Variables

#### Outcome Variable—oral impacts on daily performance (OIDP)

The primary outcome in this study was OHRQoL, measured using the Norwegian version of the OIDP questionnaire (24). This includes eight questions assessing how often over the past six months the participants, due to issues with their teeth or dentures, have experienced problems with: (1) eating and enjoying food, (2) speaking and pronouncing clearly, (3) cleaning teeth, (4) smiling or showing their teeth without embarrassment, (5) sleeping or relaxing, (6) maintaining an emotional state of well-being, (7) enjoying company of other people, and (8) carrying out daily tasks or work. Participants responded using a five-point scale with the categories: (0) never affected, (1) less than once a month, (2) once or twice a month, (3) once or twice a week, (4) every or nearly every day.

The “OIDP sum score” was calculated by summing responses to each question (range 0–32). Additionally, the responses to each item were dichotomized into “No problems” (category 0) and “Problems” (category 1–5) and summarized to a variable named “OIDP number of problems” (range 0–8). This was further dichotomized into the variable “OIDP problem” coded 0 = no problems with any item and 1 = problem with at least one item. This approach was chosen to distinguish participants with any reported impact on daily life from those reporting none, thereby providing a clinically interpretable outcome and facilitating logistic regression analyses in a relatively small sample. The use of dichotomized OIDP measures is also consistent with previous studies, where the outcome has been operationalized as the presence or absence of oral impacts on daily life (25–28).

#### Explanatory variables

***Sex and age*** were from the questionnaire. For descriptive analysis, age was categorized into the following groups: 19–29 years, 30–39 years, 40–49 years, and ≥50 years. For statistical analysis, age was used as a continuous variable.

***Education:*** Participants were asked about the highest level of education they had completed with the options: 1. Elementary school, 2. Upper secondary school or vocational education, 3. College/university less than four years or 4. College/university 4 years or more. For statistical analyses, options 3 and 4 were merged into one variable named “Higher education”.

***Financial situation:*** Participants evaluated their financial situation with the options: 1. Very good, 2. Good, 3. Average, 4. Difficult, 5. Very difficult. For statistical analyses, the responses were recategorized into: 1. Good (original options 1 and 2), 2. Average (original option 3), 3. Difficult (original option 4 and 5).

***Tobacco:*** Participants were asked if they smoked with the options: 1. No, never, 2. Before, but not anymore, 3. Sometimes, and 4. Daily. For statistical analyses, options 3 and 4 were merged into Current smoker.

***Tooth brushing frequency:*** Participants were asked how often they brush their teeth with the options: 1. Less than once a week, 2. A few times a week, 3. Once a day, and 4. Twice a day or more. For descriptive analysis, option 1 and 2 were merged into Less than daily. For regression analysis, options 1, 2 and 3 were merged into 1) Less than 2/day.

***Alcohol consumption:*** The validated Audit-C (29–31) screening tool was used to identify problematic alcohol use. Participants reported both the frequency of alcohol intake and average number of units consumed over the past 12 months. Maximum score was 12. The Audit-C scores were categorized into Low (0–3), Moderate (4–7), and High (8–12).

***Substance use:*** Participants were asked if they used drugs or addictive medications, other than alcohol, with the options: 1. Never, 2. Have tried sometimes, 3. Once a month or less, 4. 2–4 times a month, 5. 2–3 times a week, and 6. 4 times a week or more and 7. Check if you mean during relapses, or in certain periods. For descriptive analysis, these options were recategorized into the following: 1. Never (original option 1), 2. Sometimes/often (original options 2, 3, 4, 5 and 6), and 3. Relapse (original option 7). Type of drugs was registered as free text. For regression analysis, the variable was recategorized into: 1. Never (original option 1), and 2. Sometimes/Often (original options 2, 3, 4, 5, 6 and 7).

***Psychological distress:*** Symptoms of anxiety and depression were assessed with the 10-item Hopkins Symptom Check List (HSCL-10) (32). The responses from the HSCL-10 were summarized and analyzed as a continuous variable in both regression and mediation analyses.

***PQ-16 score:*** The 16-item Prodromal Questionnaire (PQ-16) (33–35) is a 16 item instrument most often used in non-hospital settings to examine psychosis risk. In the present inpatient population, where many participants have established psychotic disorders, the PQ-16 was used to capture the current level of psychotic symptoms rather than prodromal risk. Responses to PQ-16 were summarized. For descriptive analysis, this sum score was categorized as 1. 0–5 symptoms and 2. ≥6 symptoms. For regression analysis and mediation analysis, the sum-score was used as a continuous variable.

***Xerostomia:*** Participants were asked if they felt bothered by dry mouth with the options; 1. Not at all, 2. A little bothered, 3. Quite bothered, 4. Very bothered. The responses were dichotomized into 1. No/little feeling of dry mouth (original option 1 and 2) and 2. Moderate/strong feeling of dry mouth (original option 3 and 4). For the mediation analyses, xerostomia was treated as an ordinal variable (1–4) reflecting symptom severity and entered as a continuous mediator in the PROCESS analyses.

***Self-reported general health:*** Participants were asked to describe their general health with the following options: 1. Very good, 2. Good, 3. Neither/Nor, 4. Poor, 5. Very poor. For descriptive cross tabulation, the answers were recategorized into 1. Very good/good (original options 1 and 2), 2. Average (original option 3) and 3. Very poor/poor (original options 4 and 5). For regression and mediation analysis, original options 1 and 2 were merged into Very good/good and options 3, 4 and 5 were merged into Average/very poor/poor.

***Orofacial pain*:** Participants were asked three questions regarding orofacial pain and discomfort (36, 37): 1. Do you experience pain in the temples, face, jaw or jaw joint once a week or more often? 2. Does it hurt when you open your mouth wide or chew once a week or more often? 3. Do you experience your jaw locking or getting stuck once a week or more often? All questions were answered with yes/no. If at least one of the three answers were yes, the variable was coded as “Pain/Discomfort”. If all answers were no, the variable was coded as “No pain/discomfort”. For the mediation analyses, orofacial pain was treated as an ordinal variable reflecting cumulative symptom burden, operationalized as the sum of the three pain-related items (0–3), defined as the number of endorsed items (0 = no symptoms, 3 = three endorsed items). The variable was entered as a continuous mediator in the Process analyses.

#### Clinical variables

The 3rd molars were excluded from the analysis, (defining a complete dentition as 28 teeth). Caries, restorations, and missing teeth were recorded based on clinical examinations, radiographs, and clinical photographs as previously described (23).

***Decayed teeth (DT)*** Caries (primary and secondary) was graded from 1 to 5, where 1 indicated caries in the outer half of enamel, 2 caries in the inner half of the enamel, 3 caries extending into the outer third of the dentin, 4 into the middle third of the dentine, and 5 into the inner third of the dentin. In this study, we defined a tooth as decayed (DT) if it was graded with caries 3–5 (manifest caries reaching the dentine). For all statistical analyses, DT was operationalized as a continuous variable representing the number of decayed teeth per participant.

***Missing teeth (MT)*** were registered regardless of cause of loss. For all statistical analyses, MT was operationalized as a continuous variable representing the number of missing teeth per participant.

***Filled teeth (FT)*** included teeth with all types of permanent restorations, including crowns and bridge abutments. If a filled tooth had caries reaching the dentine, it was considered decayed.

#### Conceptual categorization of variables

**Figure 1:**
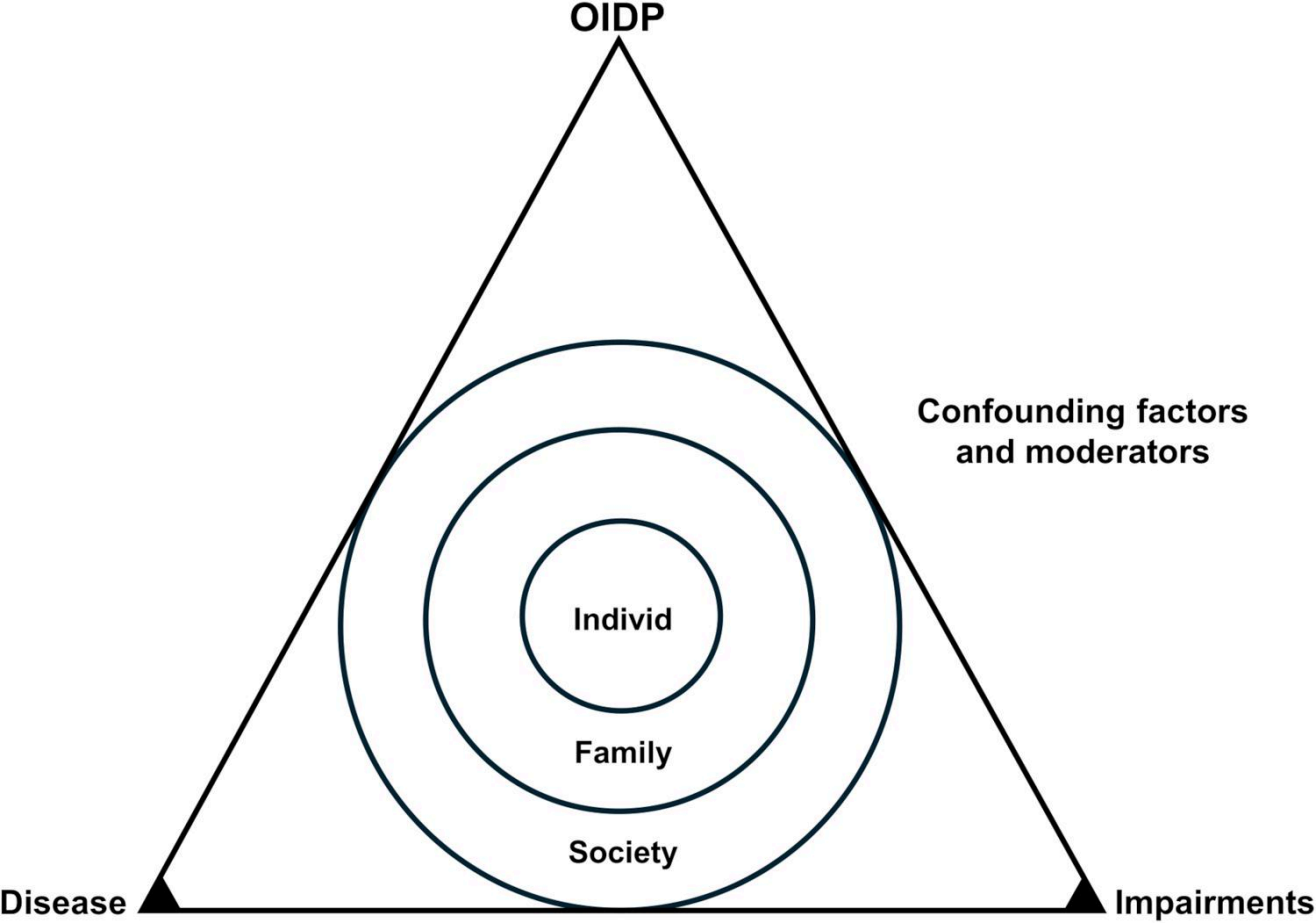
The model illustrates how multiple pathways, both direct and indirect, contribute to oral impacts on daily performance. The triangle represents the progression from “Disease” to “Impairments” (base) and “Functional Limitation/Disability” (apex, OIDP). Concentric circles reflect individual, family, and societal influences, while confounding and moderating factors along the triangle's sides highlight personal and environmental variables shaping disease processes and functional impacts.

Based on the conceptual model derived from WHO's ICIDH/ICF framework (18, 19), Fisher-Owens multilevel model (20), and Locker's adaptation (21), study variables were grouped according to a hierarchical disease–impairment–functional limitation structure. Psychological distress (HSCL-10), PQ-16 score (PQ-16) and general health (GH) were treated as disease-level variables. While HSCL-10 and PQ-16 represent specific symptom dimensions, GH was included at the same conceptual level as a broader indicator of overall health status and underlying disease burden. Oral clinical indicators (decayed teeth, missing teeth, orofacial pain and xerostomia) were classified as impairments, representing structural or functional oral disturbances. OIDP was used as the functional limitation outcome, reflecting the impact of oral conditions on daily activities. This categorization guided the analytical strategy and formed the basis for the mediation models tested in the study. To situate the empirical findings within this broader theoretical context, a conceptual model was developed to synthesize the key frameworks underlying the study (Figure 1).

### Statistical analysis

IBM SPSS Statistics version 29.0.0.0 (241) was used for statistical analyses, employing descriptive statistics and cross-tabulations.

The outcome variable, OIDP, was presented as descriptive frequency data. Continuous explanatory variables were reported as medians with 25 and 75 percentiles due to non-normal distribution. To evaluate differences in continuous variables across OIDP groups (no problems vs. problems), the non-parametric Mann–Whitney and Kruskal–Wallis tests were employed. Chi-square tests were used for categorical variables.

Analyses were conducted using complete case analysis (listwise deletion), whereby cases with missing data on variables included in each analysis were excluded. The proportion of missing data was low (<3% for all variables; range 0.7%–2.9%).

Univariate analyses were conducted as a preparatory step for factor and mediation analyses. Categories with small numbers of participants were merged to ensure adequate cell sizes and statistical stability in subsequent regression analyses. Recategorization was guided by conceptual similarity and by similarity in distributions across outcome groups, to minimize sparse data bias and improve the robustness of the estimates.

Logistic regression was applied for the dichotomous outcome of OIDP. As a sensitivity analysis, OIDP was also analyzed as a count variable using negative binomial regression. The main predictors identified in the logistic regression models were consistent across both approaches.

Building on these preparatory analyses, variable selection was guided by previous literature on oral health-related quality of life (38–40). The dataset's underlying structure and the potential overlap among the selected symptoms and substance use measures were assessed using an exploratory factor analysis (EFA) with Principal Axis Factoring and Oblimin rotation.

To examine whether the associations between GH, HSCL-10, and PQ-16 and OHRQoL were consistent with indirect pathways through clinical oral conditions, a series of simple mediation analyses was conducted using the PROCESS Macro for SPSS (version 4.2; Hayes) (41). Model 4 was applied to estimate total, direct, and indirect effects between predictors, mediators, and the dichotomous outcome.

The outcome variable in all models was OIDP, coded dichotomously (0 = no problems, 1 = problems). Accordingly, logistic regression was implemented by PROCESS. GH was included as a dichotomous predictor, whereas HSCL-10, PQ-16, DT, and MT were entered as continuous variables. Orofacial pain (0–3; number of endorsed items) and xerostomia (1–4; increasing symptom severity) were operationalized as ordinal variables and entered as continuous mediators in the PROCESS analyses. The variables GH, HSCL-10, and PQ-16 were examined in separate mediation models as predictors (X), with DT, MT, orofacial pain, and xerostomia specified as mediators (M). Age was included as a covariate in all models and adjusted for in both the a-path (X → M) and the b-path (M → ).

Indirect effects were estimated using bootstrapping with 5,000 resamples, and statistical significance was evaluated using bias-corrected 95% confidence intervals (CI). An indirect effect was considered statistically significant when the bootstrap CI did not include zero.

For mediator models (X → M), unstandardized regression coefficients were estimated. For DT and MT, a-path associations were non-significant and are therefore indicated as non-significant (N.S.) in the figures, whereas coefficients for ordinal mediators are shown explicitly. For outcome models (M → Y and X → Y), results are presented as odds ratios (ORs) with 95% confidence intervals. Model diagnostics, including goodness-of-fit statistics (McFadden's *R*^2^, Cox–Snell *R*^2^, and Nagelkerke *R*^2^), were examined to assess model fit.

Assumptions relevant to the regression models were evaluated prior to the analysis. Multicollinearity among predictors and mediators was assessed using variance inflation factors (VIF), with all values below 2, indicating no problematic collinearity. Linearity in the logit was examined for all continuous variables included in logistic regression paths (age, HSCL-10, PQ-16, DT, and MT) using the Box–Tidwell procedure. This assumption was supported for all variables. Ordinal variables with few categories (e.g., orofacial pain and xerostomia) were treated as continuous and were not subjected to formal linearity testing. No influential outliers were detected based on inspection of standardized residuals, Cook's distance, and leverage values. Overall, no major violations of model assumptions were detected.

No formal correction for multiple testing was applied. Although the analyses were guided by predefined models, they may still be considered exploratory in nature. Results were interpreted with caution, given the potential for type I error.

### Ethics

The study was approved by the Regional Committee for Medical and Health Research Ethics (approval reference number 240987, dated 16.06.2021) and the Norwegian Centre for Research Data (approval reference number 119768, dated 12.08.2021). Written informed consent was obtained from all participants.

## Results

The study included 138 participants with a mean age of 38.0 years (range: 19–70). Of these, 83 participants (60.1%) were men and 55 (39.9%) were women.

### Oral impact on daily performance (OIDP)

Table 1 presents the frequency of problems with the daily activities assessed with the OIDP questionnaire as separate items as well as sum scores.

**Table 1:** Oral impact on daily performances—frequency distribution.

	Never	<1/month	1–2/month	1*–*2/week	Almost daily or daily	Ever^a^
	*n* (%)	*n* (%)	*n* (%)	*n* (%)	*n* (%)	*n* (%)
Eat and enjoy food	62 (45.6)	6 (4.4)	10 (7.4)	26 (19.1)	32 (23.5)	74 (54.4)
Speak, pronounce	106 (77.4)	5 (3.6)	5 (3.6)	8 (5.8)	13 (9.5)	31 (22.6)
Clean teeth	83 (60.6)	4 (2.9)	10 (7.3)	20 (14.6)	20 (14.6)	54 (39.4)
Smile/show teeth	83 (61.0)	2 (1.5)	5 (3.7)	12 (8.8)	34 (25.0)	53 (39.0)
Sleep and relax	88 (64.2)	7 (5.1)	13 (9.5)	15 (10.9)	14 (10.2)	49 (35.8)
Emotionally stabile	100 (73.5)	4 (2.9)	9 (6.6)	14 (10.3)	9 (6.6)	36 (26.5)
Enjoy company	97 (71.9)	1 (0.7)	10 (7.4)	9 (6.7)	18 (13.3)	38 (28.1)
Daily task	120 (88.9)	0 (0.0)	5 (3.7)	6 (4.4)	4 (3.0)	15 (11.1)
OIDP Sum—Frequency Of Any Problem^b^	38 (27.7)	5 (3.6)	8 (5.8)	29 (21.2)	57 (41.6)	99 (72.3)

Distribution of response percentages regarding the extent to which oral conditions affect the respondents in daily life.

a “Ever” The proportion who did not respond “never” (<1/month through almost daily).

b Proportion Of participants reporting problems with at least one of the eight OIDP items at the specified frequency.

The most reported issue was difficulty eating and enjoying food, with 54.4% of participants experiencing this at least once a month, and 23.5% experiencing it daily or almost daily. Nearly 40% reported problems with cleaning their teeth or showing their teeth without embarrassment, the latter being the problem that most participants reported on a daily or almost daily basis (25.0%). Problems sleeping and relaxing were reported by 35.8%, 21.1% had problems at least once a week. The least reported issue was difficulty performing daily tasks/work, which affected 11.1% of participants.

Overall, 72.3% of participants reported difficulties with at least one of the assessed activities, and 60.0% faced one or several such difficulties at least once a week. Daily or almost daily problems were reported by 41.6% (Table 2). Among those with psychiatric disorders and/or substance use disorders who reported problems, the mean “OIDP number of problems” affecting daily life was 3.5 (range 1–8). The mean OIDP sum score, (ranging from 1 to 32) was 10.8 (SD = 7.6) among those who reported OIDP problems.

**Table 2:** Oral impact on daily performances by participant characteristics.

	All	OIDP	OIDP	*P*
		No problems	Problems	
	*n* (%) (column)	*n* (%) (row)	*n* (%) (row)	
All	137 (100.0)	38 (27.7)	99 (72.3)	
Sex				0.205^b^
Women	55 (39.9)	12 (21.8)	43 (78.2)	
Men	82 (60.1)	26 (31.7)	56 (68.3)	
Age group				0.132^b^
19–29	52 (37.2)	11 (21.6)	40 (78.4)	
30–39	27 (19.7)	7 (25.9)	20 (74.1)	
40–49	33 (24.1)	8 (24.2)	25 (75.8)	
Above 50 years	26 (19.0)	12 (46.2)	14 (53.8)	
Education				**0**.**006**^b^
Elementary school	49 (36.6)	9 (18.4)	40 (81.6)	
High school/vocational	68 (50.7)	19 (27.9)	49 (72.1)	
Higher education	17 (12.7)	10 (58.8)	7 (41.2)	
Financial situation				0.053^b^
Good	32 (23.9)	11 (34.4)	21 (65.6)	
Average	51 (38.1)	18 (35.3)	33 (64.7)	
Difficult	51 (38.1)	8 (15.7)	43 (84.3)	
General health				**0**.**002**^b^
Very good/good	58 (42.3)	25 (43.1)	33 (56.9)	
Average	38 (27.7)	7 (18.4)	31 (81.6)	
poor/poor	41 (29.9)	6 (14.6)	35 (85.4)	
Smoking				**0**.**049**^b^
Never	18 (13.1)	8 (44.4)	10 (55.6)	
Previous	43 (31.4)	15 (34.9)	28 (65.1)	
Current	76 (55.5)	16 (34.9)	61 (80.3)	
Alcohol consumption				0.213^b^
Low	58 (43.3)	14 (24.1)	44 (75.9)	
Moderate	34 (25.4)	13 (38.2)	21 (61.8)	
High	42 (31.3)	9 (21.4)	33 (78.6)	
Substance use				0.691^b^
Never	45 (33.3)	14 (31.1)	31 (68.9)	
Sometimes/Often	63 (46.3)	15 (23.8)	48 (76.2)	
Relapse	28 (20.6)	8 (28.6)	20 (71.4)	
Psychological distress				**0**.**003**^b^
No/low ≤2.35	73 (54.1)	27 (37.0)	46 (63.0)	
Moderate/high >2.35	62 (45.9)	9 (14.5)	53 (85.5)	
PQ-16 score				**<0**.**015**^b^
0–5 symptoms	82 (60.7)	28 (34.1)	54 (65.9)	
≥6 symptoms	53 (39.3)	8 (15.1)	45 (84.9)	
Xerostomia				**0**.**026**^b^
No/little	92 (67.2)	31 (33.7)	61 (66.3)	
Moderate/severe	45 (32.8)	7 (15.6)	38 (84.4)	
Tooth brushing				**0**.**010**^b^
<1/day	33 (24.3)	3 (9.1)	30 (90.9)	
1/day	31 (22.8)	8 (25.8)	23 (74.2)	
≥2/day	72 (52.9)	27 (37.5)	45 (61.5)	
Orofacial pain				**<0**.**001**^b^
No pain/discomfort	62 (45.3)	27 (43.5)	35 (56.5)	
Pain/discomfort	75 (54.7)	11 (14.7)	64 (85.3)	
**DT** Median (25-, 75 perc)	3.0 (1.0–7.0)	1.0 (0.0–5.0)	4.0 (1.5–8.0)	**0**.**002**^a^
**FT** Median (25-, 75 perc)	7.0 (4.0–11.0)	7.0 (5.0–11.0)	7.0 (4.0–10.8)	0.377^a^
**MT** Median (25-, 75 perc)	1.0 (1.0–7.0)	0.0 (0.0–2.0)	1.0 (0.0–4.0)	**0**.**024**^a^

Bold annotations mark significant findings. *P* shows the statistical significance assessed by

a Non-Parametric test: Mann U-Whitney/Kruskal Wallis or

b Pearsons Chi Square.

OIDP, Oral impact on daily performance; DT, Decayed teeth; FT, Filled teeth; MT, Missing teeth.

The prevalence of OIDP problems varied significantly across several cohort characteristics, as detailed in Table 2. Problems with at least one of the daily activities assessed with the OIDP questionnaire were relatively common among participants without higher education, those with average or poor self-rated general health, current smokers, participants with high scores for psychological distress or risk of psychosis, those experiencing xerostomia or orofacial pain/discomfort, and those who brushed their teeth less than daily. Participants reporting problems also had higher numbers of DT and MT than those reporting no problems (Table 2).

### Factor analysis

Exploratory factor analysis supported a three-factor solution based on the scree plot and interpretability, reflecting psychological distress (HSCL-10), PQ-16 score (PQ-16), and alcohol use (AUDIT-C).

The psychological distress factor comprised all HSCL-10 items, along with the GH-item and one PQ-16 item (“lost interest”), all loading above 0.45. Given its conceptual and empirical overlap with psychological distress, GH was retained for subsequent mediation analyses. The PQ-16 factor included the core PQ-16 items, with strong loadings (≥0.50) and clear separation from the other dimensions. Alcohol use items (frequency, quantity, and binge drinking) loaded consistently on a single factor. Internal consistency was good to very good across factors (HSCL-10: *α* = 0.886; PQ-16: *α* = 0.895; alcohol use: *α* = 0.806).

### Univariate regression analysis

In logistic regression, reporting OIDP problems was significantly associated with younger age, educational level, general health, psychological distress, PQ-16 score, xerostomia, orofacial pain, smoking, toothbrushing frequency, and higher numbers of decayed and missing teeth (Supplementary Table S1).

Alcohol and substance use were not included in the mediation models, as no significant direct association with OIDP was observed in the univariate analyses.

### Mediation analysis

**Figure 2:**
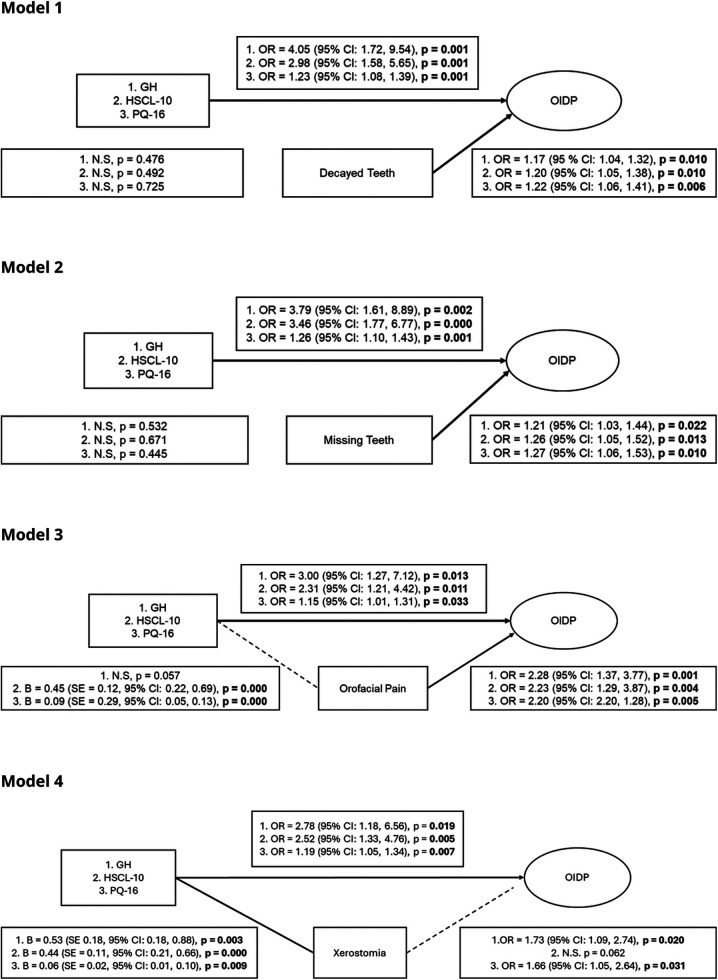
Model 1–4. Significant results are highlighted with bold arrows for pathways and bold text for key elements. Simple mediation models (PROCESS Model 4) examining the associations between general health (GH), psychological distress (HSCL-10), and PQ-16 score (PQ-16) and oral impacts on daily performance (OIDP), with decayed teeth (DT) (model 1), MT (model 2), orofacial pain (model 3) and xerostomia (model 4) as potential mediators. Odds ratios (ORs) with 95% confidence intervals are shown for significant paths. Predictors were tested in separate models, all adjusted for age.

The results from the mediation analyses are presented as path diagrams in Figure 2 (Models 1–4), illustrating the direct and indirect pathways between predictors, mediators, and OIDP. Figure 2, Model 1 showed that GH, HSCL-10, and PQ-16 were directly associated with OIDP, while none of these variables were associated with DT. Accordingly, DT were not consistent with indirect pathways between health and mental health-related variables and OIDP. However, DT were independently associated with OIDP, indicating that poor dental status was associated with daily functional impacts, independent of general health and mental health–related pathways (OR range: 1.17–1.22).

Figure 2, Model 2 similarly showed that GH, HSCL-10, and PQ-16 were directly associated with OIDP, while none of these variables were associated with MT. Accordingly, MT were independently associated on OIDP (OR range: 1.21–1.27). Structural oral disease, whether DT or MT, thus appeared to be associated with OIDP through pathways parallel to general and mental health rather than through indirect pathways.

Figure 2, Model 3 showed that HSCL-10 and PQ-16 were directly associated with OIDP and through indirect pathways involving orofacial pain, suggesting patterns consistent with partial and exposure-specific indirect associations. In contrast, GH was not associated with orofacial pain but remained directly associated with OIDP (OR: 2.28), suggesting that pain-related indirect pathways were more relevant for psychological symptom dimensions than for general health (OR range: 2.20–1.23).

Figure 2, Model 4 showed that general health, HSCL-10, and PQ-16 were directly associated with OIDP, and that all three predictors were associated with xerostomia. Xerostomia, in turn, was directly associated with OIDP in the models including general health and PQ-16, but not HSCL-10. Accordingly, indirect pathways through xerostomia were observed for general health and PQ-16 (OR range: 1.66–1.73), suggesting patterns consistent with partial indirect associations that was not observed across all predictors, whereas no such pattern was evident for HSCL-10.

## Discussion

This study demonstrates that individuals with severe psychiatric and/or substance use disorders experience a high burden of oral health problems that extend beyond physical discomfort and are associated with functional, psychological, and social dimensions of daily life. Functional limitations, such as difficulties with eating, may contribute to poor nutritional intake or food avoidance, already a concern in populations with psychiatric (42, 43) or substance use disorders (44). Problems with tooth cleaning, one of the most frequently reported disabilities, may accelerate oral disease progression, reinforcing a cycle of pain and further deterioration. Difficulties with smiling or showing teeth may reduce self-esteem and social confidence, potentially contributing to withdrawal and impaired interpersonal connections (9). Additionally, disrupted sleep and relaxation, social interactions, and emotional stability, all commonly reported problems, can further exacerbate psychiatric symptoms and reinforce social withdrawal (9). Among those with psychiatric disorders and/or substance use disorder who reported problems, the average number of daily activities affected by oral problems was 3.5, compared to 0.4 in the general Norwegian adult population (38). Moreover, the prevalence of OIDP problems in the study population was substantially higher than that recently reported in the general middle-aged population in Northern Norway (72.3% vs. 21.2%) (45). The findings are also consistent with a study from Western Norway (46) comparing individuals with severe mental illness to the general population, which likewise reported markedly higher levels of OIDP-related impacts on daily life, underscoring the extent of oral health disparities in this patient group.

The observed associations between psychological symptoms and impaired OHRQoL may also be influenced by shared behavioral risk factors. Individuals with psychiatric and/or substance use disorders are more likely to have poor oral hygiene, high sugar consumption, and be smoking, all of which are known to adversely affect oral health. These factors may partly explain the observed associations and operate alongside the pathways examined in the present study.

In addition, medication may represent an important underlying mechanism. Polypharmacy is common in this population, and many psychotropic medications are associated with xerostomia due to reduced salivary secretion. In this context, xerostomia may partly capture the oral effects of medication use in the present analyses. At the same time, medications may also be related to oral health indirectly through pathways not fully accounted for in the model, such as increased appetite, dietary changes, or reduced motivation for self-care. However, there may be confounding between medication side effects and underlying psychiatric symptoms, as symptoms of the disorder itself, particularly negative symptoms such as apathy and anhedonia in conditions like schizophrenia, may be interpreted as side effects rather than manifestations of the underlying disorder. These mechanisms may therefore contribute to the observed associations between mental health variables and OHRQoL.

Problems with at least one of the daily activities assessed with the OIDP questionnaire were relatively common among participants without higher education. Education was excluded from the regression analysis due to the small proportion of participants with higher education, which limited its statistical contribution. Nonetheless, the univariate association between education level and oral health problems, aligns with existing literature linking higher educational attainment to better physical and mental health (36). Evidence from epidemiological and clinical studies indicates that sever psychiatric disorders and substance use contribute to a cumulative life-course process characterized by disrupted educational trajectories and impaired functioning (6, 7, 47). In this context, limited educational attainment may reflect broader structural vulnerability that shapes both health-related behaviors and clinical oral health outcomes. Consistent with this life-course perspective, problems with daily activities measured by OIDP were more prevalent among participants reporting poor general health, smokers, those with high psychological distress or PQ-16 score, as well as those experiencing xerostomia or orofacial pain. In addition, OIDP impacts were associated with poorer dental status, reflected by higher numbers of decayed and missing teeth, and with less frequent tooth brushing. In this context, functional impairments such as xerostomia, decayed and missing teeth, and orofacial pain may represent potential pathways through which oral conditions are associated with daily functioning and participation. Consistent with this, previous inpatient research among individuals with substance use disorders has shown that loss of everyday activities is strongly associated with reduced well-being, emphasizing the role of functional decline beyond clinical disease markers (48). Together these findings underscore the importance of a holistic approach to oral health that integrates educational, behavioral, psychological, and clinical factors.

Age emerged as an important confounder in the relationship between clinical oral health status and OIDP. Older participants had more missing teeth, a factor strongly associated with OIDP and well documented in the literature (38, 49, 50). In contrast, younger individuals had fewer missing teeth but reported a higher prevalence of OIDP problems, as shown in a previous analysis of the same study population (23). Similar patterns have been reported elsewhere, where older adults perceive fewer oral health–related impacts despite poorer clinical status (51).

### Integration with theoretical frameworks

The findings in this study aligned closely with the theoretical models, which conceptualized OHRQoL as the endpoint of a progression from disease via impairments to functional limitations or disabilities. Within this framework, psychological distress, PQ-16 score, and general health functioned as disease-level factors, while oral conditions such as DT, MT, orofacial pain, and xerostomia represented impairments.

Across all four models, psychological symptoms and general health showed consistent direct associations with OIDP, consistent with the theoretical view that disease-level psychological factors may be related to disability through behavioral, cognitive, and psychosomatic pathways. Structural oral disease, reflected by decayed and missing teeth, did not show evidence of indirect associations between psychological symptoms, general health, and OIDP, but were independently associated with daily functioning. This places structural dental impairments as factors associated with oral disability through pathways parallel to mental health, rather than as intermediaries. In contrast, symptom-based impairments such as orofacial pain and xerostomia showed patterns consistent with indirect associations with OIDP in selected models and exposure-outcome relationships. These findings suggest that perceptual oral symptoms may partially link psychological distress and general health to oral health–related disability, although mediating effects were not consistently observed across all predictors.

Taken together, the results indicated that different types of impairments played distinct roles within the disease, impairment, and disability pathway. Structural disease did not explain the link between psychiatric disease and OIDP, whereas subjective symptom-based impairments such as pain and xerostomia did. This distinction reinforced the psychosomatic dimension of the theoretical framework illustrating that impairments with strong perceptual and sensory components may be more likely to be involved in pathways linking psychological factors and OHRQoL. Furthermore, the findings highlight the importance of addressing both disease-level factors and impairments within a holistic framework and line up with the theoretical model's emphasis on the interconnectedness of biological, psychological, and social dimensions of health.

### Strength and limitations

The study population represents a hard-to-reach and under-researched group. Individuals with severe psychiatric and/or substance use disorders are rarely represented in population-based studies, and inpatient settings may represent one of the few feasible opportunities to obtain clinically relevant data from this group. In this context, participants consisted of inpatients receiving specialized psychiatric and substance use disorder treatment. Admission to inpatient care reflects severe clinical impairment, typically involving an inability to care for oneself and/or risk of harm to oneself or others. Consequently, the sample represents individuals with more severe psychiatric and substance-related conditions than those commonly seen in outpatient settings, such as individuals with mild to moderate anxiety or depressive symptoms. The findings should therefore be interpreted in light of this high level of clinical severity and may not be generalizable to individuals with milder conditions, those receiving outpatient care, or the general population.

In addition, some degree of selection bias within the inpatient population cannot be excluded. Participation required clinical stability and the ability to provide informed consent, which may have resulted in the underrepresentation of the most severely affected individuals.

The cross-sectional design precludes conclusions about temporal relationships or causality. Although the mediation models were theoretically informed, the direction of associations cannot be established, and the findings should be interpreted as indicative of potential pathways rather than causal mechanisms.

Furthermore, The PQ-16 (33–35) is designed to screen individuals for early signs of psychosis or risk of developing psychosis and has been validated against CAARMS outcomes (Comprehensive Assessment of At-Risk Mental States), a widely used clinical tool for identifying individuals at ultra-high risk (UHR) for psychosis as well as those with manifest psychotic disorder. As PQ-16 assesses the presence rather than the severity or intensity of psychotic experiences, it does not distinguish between psychosis risk and established psychosis. Consequently, individuals with an existing psychotic disorder may also screen positive, with diagnostic differentiation intended to occur at the subsequent interview stage. The inpatient population in the present study included a substantial proportion of individuals with severe and chronic psychiatric conditions, including established psychotic disorders. For these participants, the PQ-16 score therefore likely reflected the current level of psychotic symptomatology rather than prodromal risk. Although active psychotic symptoms may challenge an individual's ability to provide fully reflective self-reports, potentially affecting data validity and considerations related to decision-making capacity, such effects may be mitigated under controlled data-collection conditions.

In the present study, data were collected through guided interviews conducted by trained research personnel, and clinical staff assessed participants as sufficiently stable to participate. In addition, several participants were accompanied by ward personnel during the interview, which may have further supported comprehension and engagement. Taken together, these procedures likely reduced the risk of impaired understanding or compromised consent. Nevertheless, some degree of response bias related to psychopathology cannot be entirely excluded.

Standard AUDIT-C cut-offs vary by sex, with thresholds of ≥4 for men and ≥3 for women typically indicating harmful use or dependence (29, 30, 52, 53). In the present inpatient sample, however, alcohol consumption was presumed to be high across sexes, which reduced the discriminatory value of sex-specific thresholds. To better capture variation within this high-use population, AUDIT-C scores were therefore categorized uniformly into low, moderate, and high use. This approach enabled more meaningful differentiation of alcohol use patterns in the regression analyses while maintaining clinical relevance for this specific setting. Residual confounding cannot be excluded. Although several relevant variables were included in the analyses, other potentially important factors, such as detailed patterns of substance use, medication use, and duration or severity of psychiatric illness, were not fully captured and may have influenced the observed associations.

The modest sample size (*N* = 138) reflects the practical and ethical challenges of recruiting individuals with severe psychiatric and/or substance use disorders in an inpatient setting. Participation required clinical stability and the ability to provide informed consent, which limited the pool of eligible participants. The sample size also limited the range of statistical analyses and reduced statistical power, which should be considered when interpreting the findings. In particular, the sample size may have limited the statistical power to detect indirect effects in the mediation analyses, and the estimated mediation effects should therefore be interpreted with caution. A further limitation is that OIDP was dichotomized for regression and mediation analyses. Although this approach improved interpretability and analytical feasibility, it reduced variability in the outcome and may have obscured differences in the severity and frequency of oral impacts. However, it was considered appropriate given the sample size and the aim of providing a clinically interpretable outcome, and is consistent with previous Norwegian and international studies, facilitating comparability across populations.

Sensitivity analyses using negative binomial regression with OIDP as a count outcome showed largely consistent results for the main predictors, although fewer variables reached statistical significance. This likely reflects greater variability within the group reporting oral impacts and suggests that, in this population, the distinction between having any oral impact vs. none may be more informative than differences in the number of impacts.

## Conclusion

Adults with psychiatric and/or substance use disorders demonstrated a high burden of oral impacts on daily performance, affecting essential functions such as eating, oral hygiene, sleep, and social interaction. Psychological distress and PQ-16 score were consistently associated with impaired OHRQoL, while symptom-based oral impairments, particularly orofacial pain, were associated with indirect pathways in these associations. In contrast, clinically evident disease, reflected by decayed and missing teeth, was independently associated with OIDP but was not consistent with indirect pathways related to mental health variables. Future longitudinal studies are needed to clarify the temporal relationships and potential causal pathways underlying these associations.

### Clinical implications

The findings suggest that oral health should be more systematically considered in psychiatric inpatient care. Given the high prevalence of oral impacts on daily functioning, incorporating simple oral health screening into routine assessments may help identify unmet needs at an early stage. The observed associations between psychological symptoms and OHRQoL further support the need for closer collaboration between dental and mental health services. Establishing clearer referral pathways and interdisciplinary follow-up may facilitate more comprehensive and patient-centered care.

## Data Availability

The datasets generated and analyzed during the current study are not publicly available due to restrictions under the EU General Data Protection Regulation (GDPR), which prevents these datasets from being made into open access data. However, access to the de-identified data can be granted by the corresponding author upon reasonable request and is subject to strict adherence to privacy protocols. Interested researchers are encouraged to contact the corresponding author with a detailed proposal of their intended use of the data. All requests will undergo an ethical review to ensure compliance with relevant regulations and ethical standards. Requests to access the datasets should be directed to Kristina Kantola: kristina.kantola@tromsfylke.no
