# Supplementary material for: Oral health-related quality of life in adult inpatients with psychiatric and/or substance use disorders: a Norwegian cross-sectional study
Source: Front Oral Health. 2026 May 4;7:1805653. doi: 10.3389/froh.2026.1805653 (PMC13180736; doi:10.3389/froh.2026.1805653)
Supplement: Supplementary file 1 [file Table1.docx]

**Supplementary Table 1 Univariate Regression – Outcome OIDP dichotomized**

| **Variable** | **Logistic Regression**  **OIDP problems/no problems**  **OR (95% CI)** | **p** |
| --- | --- | --- |
| *Sex* |  |  |
| Female | Reference |  |
| Male | 0.60 (0.27, 1.33) | p = 0.207 |
|  |  |  |
| Age  Continuous | **0.95 (0.92, 0.98)** | **p < 0.001** |
|  |  |  |
| *Education* |  |  |
| Elementary | **6.35 (1.9,21.2)** | **p = 0.003** |
| High School/Vocational | **3.68 (1.22, 11.09)** | **p = 0.020** |
| Higher Education | Reference |  |
|  |  |  |
| *Self-reported general health* |  |  |
| Very good/good | Reference |  |
| Average/very poor/poor | **3.85 (1.75, 8.47)** | **p < 0.001** |
|  |  |  |
| *Psychological distress* |  |  |
| Continous | **3.22 (1.74 – 5.98)** | **p < 0.001** |
|  |  |  |
| *PQ-16 score* | Reference |  |
| Continuous | **1.20 (1.07, 1.34)** | **p = 0.002** |
|  |  |  |
| *Xerostomia* |  |  |
| No/little | Reference |  |
| Moderate/Severe | **2.76 (1.11, 6.89)** | **p = 0.030** |
|  |  |  |
| Orofacial Pain |  |  |
| No problems | Reference |  |
| Problems | **4.49 (1.99, 10.12)** | **p < 0.001** |
|  |  |  |
| *Smoking* |  |  |
| Never/previous | Reference |  |
| Sometimes/Current | **2.46 (1.14, 5.30)** | **p = 0.021** |
|  |  |  |
| *Alcohol* |  |  |
| Low | Reference |  |
| Moderate | 0.51 (0.21, 1.29) | p = 0.155 |
| High | 1.17 (0.45, 3.02) | p = 0.751 |
|  |  |  |
| *Substance use* |  |  |
| Never | Reference |  |
| Sometimes/often | 1.32 (0.60, 2.90) | p = 0.496 |
|  |  |  |
| *Toothbrushing frequency* |  |  |
| 2 times a day or more | Reference |  |
| 1 time a day or less | **2.89 (1.29, 6.47)** | **p = 0.010** |
|  |  |  |
| *Regular dental visits* |  |  |
| At least every second year or more | Reference |  |
| More than two years, acute, never | 1.78 (0.83, 3.82) | p = 0.138 |
|  |  |  |
| **DT**  Median (25-, 75 perc) | **1.17 (1.04, 1.30)** | **P = 0.008** |
| **MT**  Median (25-, 75 perc) | **1.17 (1.01, 1.35)** | **p = 0.038** |

***Table legend***

Univariate regression analyses examining associations between explanatory variables and oral impacts on daily performance (OIDP), analyzed as both a count outcome and a dichotomized outcome. Logistic regression was used for dichotomized OIDP (no problems vs. problems), and results are presented as risk ratios (OR) with 95% CI. Reference categories are indicated. Statistically significant associations (p < 0.05) are shown in bold. Continuous variables were analyzed per unit increase.

***Abbreviations***

OR, odds ratio; CI, confidence interval; OIDP, oral impacts on daily performance; DT, decayed teeth; MT, missing teeth.
